# Study protocol: the Australian genetics and life insurance moratorium—monitoring the effectiveness and response (A-GLIMMER) project

**DOI:** 10.1186/s12910-021-00634-2

**Published:** 2021-05-21

**Authors:** Jane Tiller, Aideen McInerney-Leo, Andrea Belcher, Tiffany Boughtwood, Penny Gleeson, Martin Delatycki, Kristine Barlow-Stewart, Ingrid Winship, Margaret Otlowski, Louise Keogh, Paul Lacaze

**Affiliations:** 1 Public Health Genomics, Department of Epidemiology and Preventive Medicine, Monash University, Melbourne, VIC Australia; 2 Murdoch Children’s Research Institute, Parkville, VIC Australia; 3 Victorian Clinical Genetics Services, Parkville, VIC Australia; 4 The University of Queensland Diamantina Institute, The University of Queensland Dermatology Research Centre, Brisbane, QLD Australia; 5 Australian Genomics, Parkville, VIC Australia; 6 Faculty of Medicine, The University of Queensland, Brisbane, QLD Australia; 7 Deakin Law School, Melbourne, VIC Australia; 8 Northern Clinical School, Faculty of Medicine and Health, University of Sydney, Sydney, NSW Australia; 9 Department of Medicine, University of Melbourne, The Royal Melbourne Hospital, Parkville, VIC Australia; 10 Genomic Medicine and Family Cancer Clinic, Royal Melbourne Hospital, Parkville, VIC Australia; 11 Faculty of Law and Centre for Law and Genetics, University of Tasmania, Hobart, TAS Australia; 12 Centre for Health Equity, Melbourne School of Population and Global Health, The University of Melbourne, Carlton, VIC Australia

**Keywords:** Genetics, Life insurance, Genetic discrimination, Moratorium, Australia, A-GLIMMER, Realist evaluation, Stakeholder engagement

## Abstract

**Background:**

The use of genetic test results in risk-rated insurance is a significant concern internationally, with many countries banning or restricting the use of genetic test results in underwriting. In Australia, life insurers’ use of genetic test results is legal and self-regulated by the insurance industry (Financial Services Council (FSC)). In 2018, an Australian Parliamentary Inquiry recommended that insurers’ use of genetic test results in underwriting should be prohibited. In 2019, the FSC introduced an industry self-regulated moratorium on the use of genetic test results. In the absence of government oversight, it is critical that the impact, effectiveness and appropriateness of the moratorium is monitored. Here we describe the protocol of our government-funded research project, which will serve that critical function between 2020 and 2023.

**Methods:**

A realist evaluation framework was developed for the project, using a context-mechanism-outcome (CMO) approach, to systematically assess the impact of the moratorium for a range of stakeholders. Outcomes which need to be achieved for the moratorium to accomplish its intended aims were identified, and specific data collection measures methods were developed to gather the evidence from relevant stakeholder groups (consumers, health professionals, financial industry and genetic research community) to determine if aims are achieved. Results from each arm of the study will be analysed and published in peer-reviewed journals as they become available.

**Discussion:**

The A-GLIMMER project will provide essential monitoring of the impact and effectiveness of the self-regulated insurance moratorium. On completion of the study (3 years) a Stakeholder Report will be compiled. The Stakeholder Report will synthesise the evidence gathered in each arm of the study and use the CMO framework to evaluate the extent to which each of the outcomes have been achieved, and make evidence-based recommendations to the Australian federal government, life insurance industry and other stakeholders.

**Supplementary Information:**

The online version contains supplementary material available at 10.1186/s12910-021-00634-2.

## Background

The use of genetic test results in risk-rated insurance is a significant concern internationally [1–4]. A major concern, based on international literature, is the deterrence of consumers from pursuing clinical genetic testing and being involved in genetic research due to insurance fears [5–12]. The use of genetic test results to discriminate against insurance applicants is a form of genetic discrimination (GD), defined as “differential treatment of asymptomatic individuals or their relatives on the basis of real or assumed genetic differences or characteristics” [13, p.64]. In response to the need to address the social and financial impacts of GD in life insurance, many countries have banned or restricted the use of genetic test results in underwriting [2]. Legislation such as Canada’s *Genetic Nondiscrimination Act* (2017) prohibits insurers (and all other entities offering goods and services) from using genetic test results without an individual’s express consent. In the US, the *Genetic Information Nondiscrimination Act* (2008) (GINA) limits the use of genetic information only in health insurance underwriting (and employment contexts). It does not apply to life insurance, although some individual states have legislated to limit genetic discrimination in life insurance [14].

Other jurisdictions have implemented alternative mechanisms, such as a moratorium in the UK (now the Code on Genetic Testing and Insurance [15]) which was introduced in 2001 as an agreement between the UK government and the Association of British Insurers [16]. Under the UK moratorium, which has no end date but is reviewed every three years, individuals applying for life policies < £500,000 are not required to disclose any genetic test results. For policies exceeding that amount, only test results pertaining to Huntington disease must be disclosed.

### Australia

In Australia, risk-rated insurance is provided by life insurers, not health insurers. Under the *Disability Discrimination Act* 1992 (Cth), life insurers are legally permitted to use genetic test results to discriminate against all applicants [1]. Use of genetic test results in life insurance underwriting is self-regulated by the insurance industry, through mandatory Standards published by the Financial Services Council (FSC), the peak body that represents the majority of life insurers in Australia. Recent Australian research highlights ongoing issues with GD in life insurance, including lack of adherence to legal requirements and industry self-regulated policies [7, 8, 17, 18].

Additionally, GD in this context has been identified as one of the most significant ethical, legal and social issues (ELSI) in genomics currently facing Australia, both in terms of policy development and its impact on genetic research and clinical services [19]. In 2018, a Parliamentary Joint Committee (PJC) recommended that the use of genetic test results in life insurance be banned in Australia [20]. The Committee’s report affirmed that GD is a problem of increasing significance (s9.86), and that based on current evidence, a duty to disclose genetic test results to life insurance companies is not appropriate (s9.84). Preventing such a duty of disclosure was considered to be more important for consumers than any concerns regarding adverse selection (which, in the Committee’s view, were overstated by the insurance industry) (s9.87–88). The Committee was concerned about at-risk individuals choosing to not have clinically-indicated genetic testing because of insurance discrimination fears, and the impact of reduced genetic research participation on Australia’s international research success (s9.89). To address these concerns, the Committee recommended that a moratorium should be urgently implemented to prohibit life insurers from using genetic test results that may predict future health concerns, and that it should take a form similar to the moratorium in the UK (s9.93). The Committee also recognised substantial concerns regarding self-regulation and its inherent conflicts of interest (s9.94), and considered that the federal government should monitor the FSC’s implementation of, and insurers’ compliance with, the moratorium, and consider implementing non-discrimination legislation if necessary (s9.96).

**Fig. 1:**
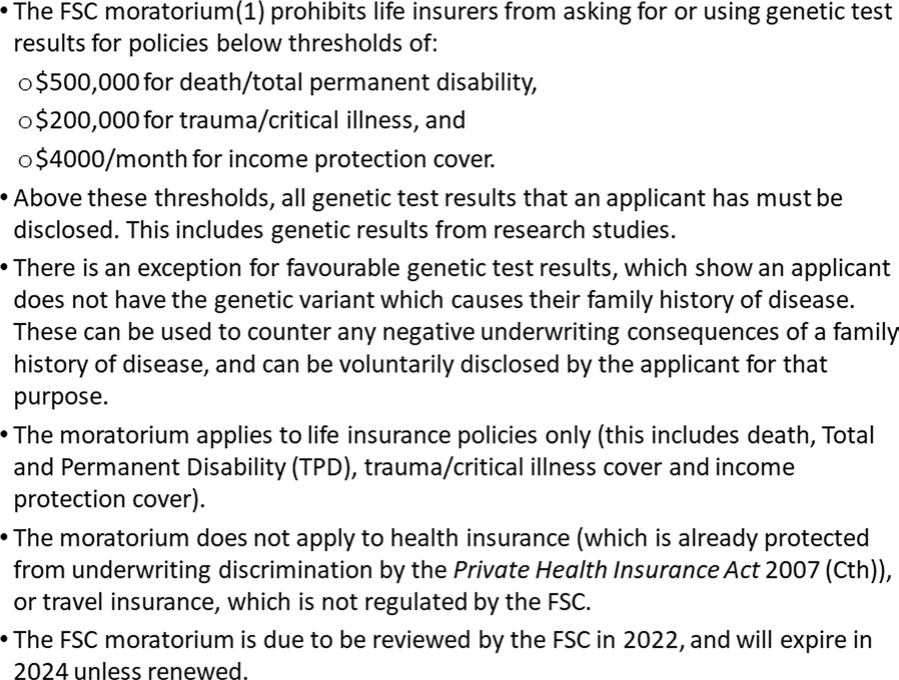
Summary of the Australian (FSC) moratorium

**Fig. 2:**
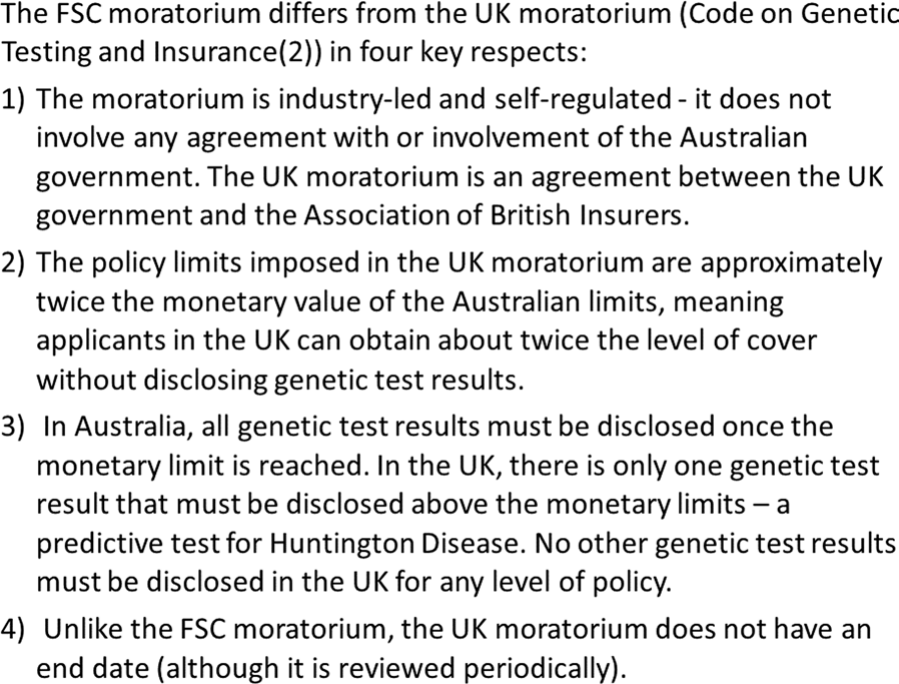
Differences between the Australian and the UK moratoria

Although the federal government has not yet responded to the recommendations, in July 2019 the FSC independently introduced an industry-led moratorium [19] restricting insurers’ use of genetic test results (see Fig. 1). This moratorium differs in four key respects from the UK moratorium (see Fig. 2). It does not change the legal position applicable to insurers under the *Disability Discrimination Act* 1992 (Cth)—that is, insurers are still legally allowed to use genetic test results to discriminate against all applicants [1]. This means that although the FSC expects its member companies to comply with the Standard containing the moratorium, it is not a legally enforceable document.

The A-GLIMMER study, funded by the Australian government’s Genomic Health Futures Mission, will evaluate the current Australian response to GD in life insurance:

RESEARCH QUESTION: To what extent does the self-regulated FSC moratorium achieve the critical policy aims identified by the Parliamentary Joint Committee (PJC)?

The aims of the recommended policy change in this area, as discussed in the PJC Report [20] are:

1. To reduce consumer fears related to insurance, which deter the uptake of clinical genetic testing and/or research participation (s9.98)
2. To eliminate genetic discrimination in the Australian life insurance industry (ss9.84 &9.86)
3. To remove a barrier currently compromising the success of genetic medicine in Australia (s9.89)
4. To ensure Australian government oversight and monitoring to combat concerns with industry self-regulation (ss 9.94 & 9.96)

Our research project will assess whether the moratorium is effective in achieving these aims. This research will serve a critical role in increasing the evidence base internationally and helping Australia achieve appropriate long-term regulation for this important issue, taking into consideration the perspective of all key stakeholders [21].

Internationally, various measures have been introduced to address GD. Research has been conducted into the effectiveness of the regulatory mechanisms used in European countries, such as ethnographic fieldwork within insurance companies [22] and postal questionnaires to individuals with a pathogenic variant [23]. Varying levels of effectiveness are reported, demonstrating the need to monitor compliance with and effectiveness of recently implemented policy changes. Although genetic discrimination concerns among genetic counsellors decreased following the US GINA’s commencement [24], non-genetic clinicians held considerably greater concerns, suggesting lower awareness in that group. A survey of cancer support group members [25] demonstrated limited understanding of GINA’s non-discrimination protections, and <20% of the general public who were surveyed were aware of GINA [26], suggesting a need for a concerted effort to educate patient populations and the general public about policy changes. Research following the UK moratorium’s introduction found that some individuals still reported difficulties obtaining insurance [27, 28], also demonstrating the need for continued research into the implementation and effectiveness of such policy changes following their introduction. No research to date has tested consumer knowledge of, or insurance experiences following the Canadian GNA’s commencement.

We have identified four major stakeholder groups, whose perspectives must be considered in order to rigorously assess whether the current Australian moratorium is an appropriate and effective long-term regulatory solution. Some research has previously been conducted internationally on these stakeholder groups to gauge experiences and perceptions of genetic discrimination, views on regulation of genetic discrimination and knowledge of relevant local non-discrimination instruments, as set out below. While these studies represent findings at various timepoints across a variety of regulatory contexts, which may differ from those currently in Australia, they demonstrate the research which has been conducted in this area.

### Consumers

Since the 1990s, numerous studies in North America, the United Kingdom, Europe and Australia have described concerns regarding GD. These concerns were voiced by at-risk clinical patients [29–39], support groups [40], and the general public [41]. Some consumers reported feeling coerced into having genetic testing to make themselves eligible for insurance or reduce premiums [42]. Several studies reported difficulty in obtaining health and/or life insurance experienced by unaffected relatives of individuals with genetic conditions [43–45], healthy adults who had tested negative for a familial pathogenic variant [44, 45], and asymptomatic individuals with a pathogenic variant who had mitigated their risk through treatment interventions and/or surveillance [27, 32, 45–50]. Although more recent legislative and other changes mean that some of the circumstances allowing these instances of GD no longer exist, these studies demonstrate the impact GD has had on consumers over a long period of time, making them a critical stakeholder group for continued research.

### Health professionals

Health professionals (HPs) —both genetic and non-genetic clinicians—are key to ensuring adequate communication of information about GD to patients. In a survey undertaken before the recent introduction of Canadian non-discrimination legislation [51], all Canadian genetic counsellors surveyed reported that they discuss insurance implications with clients. In Australia, genetic counsellors are required under the applicable professional guideline to discuss insurance implications with clients considering genetic testing where relevant [52]. Accordingly, HPs often experience firsthand the deterrent effects of GD fears on genetic testing decisions, and are often the first to hear reports of GD from patients. A US study conducted in 2000 [53], which asked genetic counsellors how they would behave if they were personally at risk of inheriting a cancer-predisposing genetic variant, was repeated in 2014 after the introduction of non-discrimination legislation [54]. It showed marked changes in perspectives following the policy change, including greater comfort with providing personal details when undergoing a test.

Various studies have also surveyed health professionals without a genetics qualification about their views and experiences regarding genetic testing and insurance discrimination. In one US study of over 1000 physicians and nurse practitioners [55], 96% of participants considered their patients would benefit from genetic testing, but 75% believed patients would not pursue testing due to GD fears. GD concerns were reported by 11% to justify non-referral of patients to genetics services. In another US study [56], 12% of genetics professionals and 14% of primary care physicians reported instances where asymptomatic patients had been denied life insurance on the basis of a genetic predisposition to disease. In Denmark, where insurers are prohibited from asking applicants about genetically determined risk of disease [57], health professionals reported that insurance concerns arose in > 5% of consultations, and led to genetic testing not proceeding in 1 in 200 cases.

Studies have also tested health professionals’ knowledge and understanding of legal non-discrimination provisions. In one US study, > 90% of participants (n = 1110) had an inaccurate knowledge of current legal protections [58], and in another, less than 35% of questions about legal non-discrimination protections were answered correctly [55]. Only 46% of Canadian pharmacists surveyed in 2018 regarding pharmacogenetics (n = 99) were aware of existing non-discrimination legislation [59].

### Genetic researchers

International researchers have described the deterrent effect of GD fears on research participation [10]. Evidence in the Canadian Senate proceedings which considered Bill S-201 (now the *Genetic Non-Discrimination Act*) showed that more than a third of families with “very sick children”, declined to participate in a free research study because of such fears [11]. Less than 7% of invited parents of sick newborn babies participated in the US BabySeq study, with some decliners citing insurance discrimination concerns as a contributing factor [12]. Similarly, 25% of decliners in the US MedSeq study (in which genetic results are stored in participants’ medical records) cited fear of insurance discrimination as the primary reason for declining [4].

### The financial industry

Research has been conducted internationally (primarily in the USA) directly with insurance companies to understand their practices and perspectives regarding the use of genetic test results in underwriting. This research includes both life and health insurance providers, but more recently has been focused on health insurers with the introduction of GINA legislation (which applies to health but not life insurers) in 2008. In 1993, medical directors of US life insurance companies were surveyed [60] using a mailed questionnaire about current practices and policies, and future perspectives, around collecting and using genetic information in underwriting. A 2012 US study [61] used online and mail-based methods to survey health insurance plan medical directors about their companies’ policies regarding, among, other things, genetic testing for individuals at risk of familial colorectal cancer syndromes. Other US studies [62, 63] asked health insurers to underwrite hypothetical insurance applicants. In one study [62] (n = 12), only three insurers had an underwriting policy related to genetic testing.

### Project rationale

It is critical that the impact, effectiveness and appropriateness of the FSC moratorium is monitored, taking into account these different stakeholder perspectives (consumers, healthcare providers, researchers and the financial services industry), to ensure that the proposed FSC review in 2022 is informed by rigorous and evidence-based submissions. Currently, there are no other mechanisms in place to do this, and this project addresses that critical gap. Our project, funded by an Australian government grant, will utilise a nationally coordinated effort to collect data from different stakeholder perspectives, to build a complete picture of the impact of the moratorium.

## Design and methods

### Methodological approach

To systematically assess the impact of the moratorium, a realist evaluation paradigm was employed in constructing the evaluation framework. “Realist evaluations asks not, ‘What works?’ or, ‘Does this program work?’ but asks instead, ‘What works for whom in what circumstances and in what respects, and how?’” [64, p.2]. The realist evaluation, which adopts a context-mechanism-outcome (CMO) approach to conceptualise interventions, is appropriate for this project, as there are multiple contexts pertaining to a range of stakeholders. By defining the specific context, mechanism and outcomes for each of the stakeholder groups, an evaluation can be designed to determine how and how well the intervention (i.e. moratorium) achieves its stated objectives. Pawson and Tilley [65] say that ‘programs work [have successful ‘Outcomes'] only in so far as they introduce the appropriate ideas and opportunities ['mechanisms'] to groups in the appropriate social and cultural conditions ['contexts'].’ The realist evaluation follows from this premise. The first step is to define the relevant outcomes (see below). The second step is to determine the relevant contexts, mechanisms and measures of these outcomes (see Table 1). Step three is to design an evaluation methodology that can test whether, how, where and to what extent each of the outcome measures represent achievement of the moratorium aims.

**Table 1:** Data collection

Intervention: genetics and insurance moratorium					
Actor	Context	Mechanisms	Outcome measures	Objectives	Data collection methods
1. Consumers	Consumer knowledge of moratorium	Widespread community promotion of the moratorium	Knowledge of existence of moratorium and accurate understanding of its terms	1.1	General population survey Genetic testers survey Pre-testers and decliners survey
	Consumer experiences	Adequate consumer protection is implemented Regulations are complied with	Increased uptake of genetic testing or reduction in delay Less distress/confusion about insurance and genetics for those testing and tested Fewer reports of adverse insurance events based on genetic data	1.2, 1.3	
2. Health Professionals (HPs)	HPs’ knowledge of moratorium	Promotion of the features of the moratorium to HPs Guidelines/processes to assist HPs to communicate with patients	Accurate knowledge of moratorium terms Confidence with explaining moratorium to patients	2.1	Health professionals survey
	HPs’ experience of patient attitudes and behaviours	Adequate consumer protection is implemented Dissemination of existence and terms of moratorium to patients	HP reports of increased uptake of genetic testing and reduced delays to testing Less distress/confusion about insurance and genetics for these testing and tested	2.2	
	HPs’ views on regulation	Adequate consumer protection is implemented	HP reports that regulation is adequate to protect patients	2.3	
3. Financial industry	Financial industry knowledge/ understanding of moratorium	Industry engagement and dissemination	Accurate understanding of moratorium terms	4.1	Financial advisor survey FSC member survey
	Financial industry implementation	Adjusted industry standards and processes	Accurate and complete recording of all instances of receiving genetic information into FSC database Reduced rate of receipt of genetic test results Reduced occurrences of adverse insurance events based on genetic test results Industry forms and processes reflect the terms of moratorium	4.2, 4.3	Analysis of industry database Application form analysis FSC Underwriters interviews or focus groups
4. Genetic research community	Researchers’ and research participants’ awareness of moratorium	Updated HREC guidelines, templates for direct communication to research participants	Increased clarity for researchers and participants, easier communication	3.1, 3.2	Researcher interviews
	Research participants’ behaviour	Adequate consumer protection is implemented	Reduced number of insurance concerns Reduced rate of research decliners due to insurance concerns	3.2	

### Outcomes

For the moratorium to accomplish its intended aims (see above), the following outcomes must be achieved:

1. Widespread and accurate awareness of the existence of the moratorium and its terms among consumer groups, health professionals, genetic researchers and research participants, ethics committees, financial industry members and regulators.
2. Confidence among consumers, health professionals, researchers and the insurance industry that the moratorium terms are strictly adhered to, and that breaches are rectified.
3. Timely and regular updates to policy, practice and processes in health care, industry and research to reflect the moratorium (e.g. industry practices, policy and processes, consent forms for genetic testing, policy and practice in genetics services and human research ethics committee (HREC) guidelines).
4. Adherence to the terms of the moratorium in the collection and use of genetic test results by all insurance companies, in practice.

Inattention to any of these areas will reduce the ability of the moratorium to achieve its intended outcomes.

### Mixed methods data collection

**Fig. 3:**
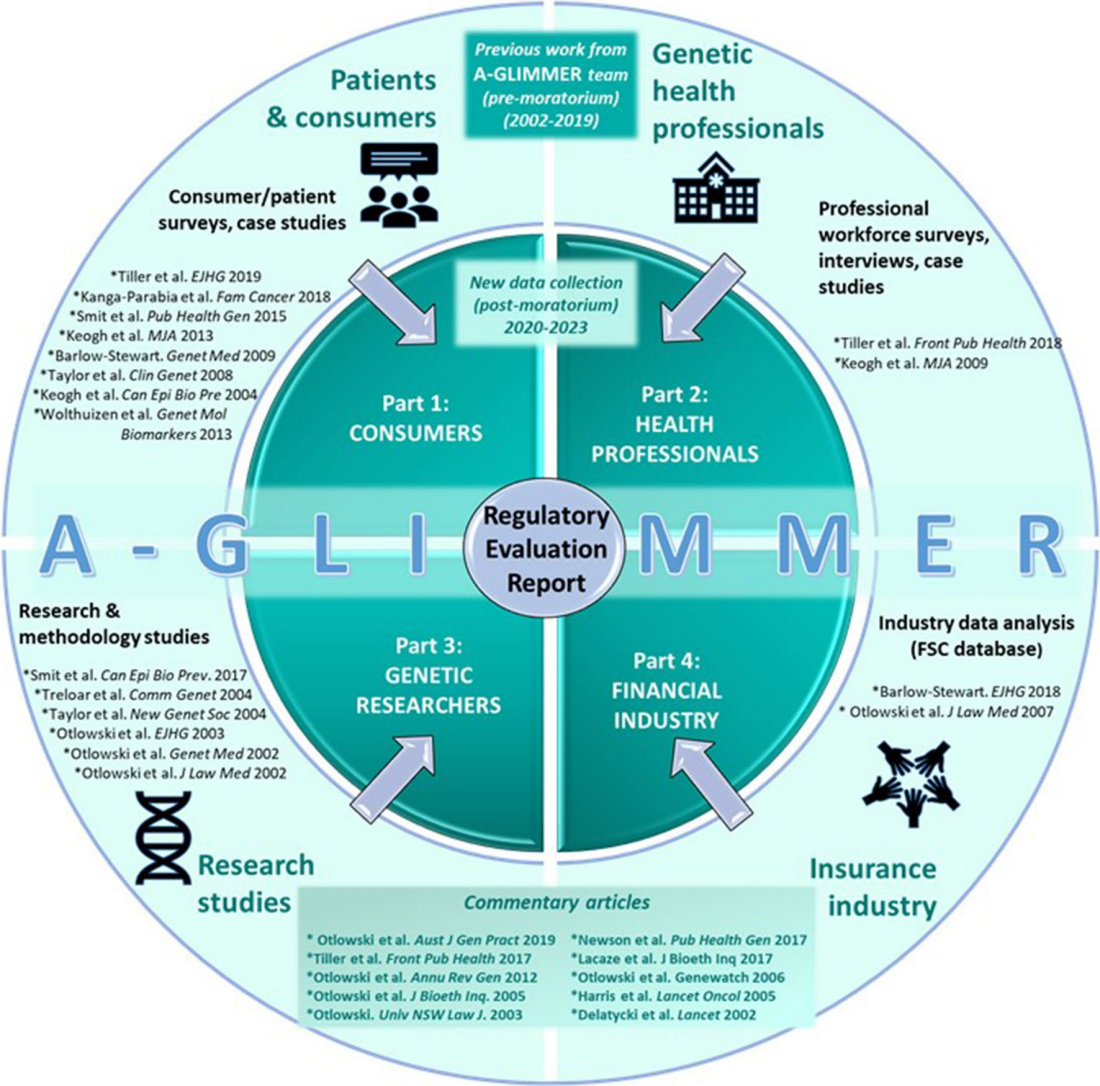
Summary of A-GLIMMER project (image created by authors)

As indicated in Table 1, a mixed methods design using both qualitative and quantitative data collection from a range of stakeholders will be used, incorporating pre- and post-moratorium comparisons where possible. No single methodological approach is capable of capturing all the data needed to evaluate the impact of this moratorium. Historically, data collection in the area of GD has proven challenging. Therefore, baseline or pre-moratorium data is incomplete and of varying quality. Where possible, relevant pre-existing research will be used to guide our methods and pre-existing measures will be used where possible to determine if the moratorium goals have been achieved. Figure 3 sets out a summary of the pre-moratorium research which has previously been conducted across the different stakeholder groups, and the research which will be conducted through the A-GLIMMER project.

In order to assess the outcome measures in Table 1, a number of objectives have been developed and a methodological approach to collect data to measure these objectives will be outlined for each stakeholder group. In part 1, we describe the research that will take place with consumers, in part 2, the research with health care professionals, in part 3, the approach we will take with researchers, and in part 4, our research program for working with the financial industry. In addition to the data collected through these mechanisms, the research team will seek out complementary data from other sources such as complaints to the Australian Financial Complaints Authority and the Australian Human Rights Commission, to enrich the data where possible.

#### Part 1: consumers

**Fig. 4:**
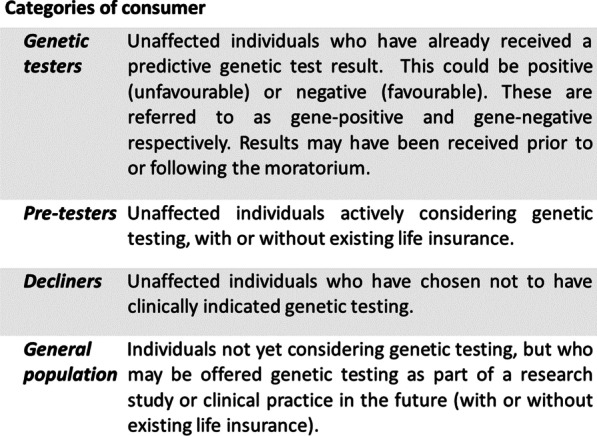
Categories of consumer

When considering genetic testing, a consumer is any individual who has had, or may have in the future, a genetic test. Consumers include those with a personal and family history of genetic or medical conditions, as well as ostensibly healthy individuals who may consider genetic testing for potential preventative health benefit or may be offered population genetic testing or genetic testing as part of a research study. With respect to genetic testing and life insurance, individuals fall into a range of different categories (see Fig. 4).

Part 1 of A-GLIMMER is designed to address the following objectives (see Fig. 4 for definitions):

- **OBJECTIVE 1.1** Assess levels of awareness and understanding of the moratorium in the *general population*, *genetic testers, pre-testers and decliners*
- **OBJECTIVE 1.2** Assess the self-described impact of the moratorium on the decision-making of *pre-testers and decliners*
- **OBJECTIVE 1.3** Assess the impact of the moratorium on *genetic testers’* ability to access insurance products compared to pre-moratorium

Prior to finalising the protocol, a meeting of consumer representatives (from disease support groups and the general community) was held to seek input regarding the proposed methodology for gathering consumer views.

##### Genetic testers survey—Objectives 1.1 and 1.3

Prior to the commencement of the moratorium, research was conducted with Australian consumers to assist with identifying experiences in access to life insurance products following genetic testing [66]. This research focussed on consumers with positive genetic test results and was limited to consumers associated with two consumer support groups—Lynch Syndrome Australia (LSA) and Pink Hope, a support organisation for people with or at risk of breast cancer-predisposing pathogenic variants. Through these groups, 174 consumers with cancer-predisposing variants were surveyed, providing baseline data on consumers’ views and experiences.

Post-moratorium, this research methodology will be repeated and expanded. Individuals with variants that increase their risk of disease, as well as favourable results that negate a family history of disease, will be surveyed to capture levels of understanding of the moratorium, impact of the moratorium on decision-making and experiences with accessing life insurance products. Recruitment will be through support groups and other consumer groups, but the reach will be expanded considerably to groups supporting consumers with a range of genetic conditions, including but not limited to LSA, Pink Hope, Mito Foundation, Familial Hypercholesterolemia Network Australia, Rare Cancers Australia, Genetic Undiagnosed and Rare Disease Network, Rare Voices Australia, and Cancer Council Victoria. With an expanded reach, we expect that the number of participants will exceed the number from the previous survey.

##### Pre-testers and decliners survey—Objectives 1.1 and 1.2

Unaffected individuals who are considering having predictive genetic testing will be surveyed to capture levels of understanding of the moratorium and the impact of the moratorium on decision-making. Decliners will be surveyed to understand reasons for their decision to not have testing.

The PRiMo (using Polygenic Risk Modification to improve breast cancer prevention) trial is recruiting female participants who will be offered genetic testing through Australian Familial Cancer Clinics (FCCs) for breast and ovarian cancer-predisposing genetic variants. Questions regarding knowledge of the moratorium, influence of insurance implications on decisions regarding genetic testing, and experiences with accessing life insurance will be included in the questionnaires received by participants soon after receiving results. Follow-up questions about experiences with accessing life insurance will be included in questionnaires administered at subsequent 6-12 month intervals.

Males attending an FCC and considering predictive genetic testing for adult-onset autosomal dominant conditions, and females considering predictive genetic testing for adult-onset autosomal dominant conditions who are not eligible for PRiMo, will be invited separately to answer questions regarding knowledge of the moratorium, influence of insurance implications on decisions regarding genetic testing, and experiences with accessing life insurance.

##### General population survey—Objectives 1.1 and 1.2

Each year, the Australian Consortium for Social and Political Research Incorporated administers the Australian Survey of Social Attitudes (AuSSA) to representative sections of the general public. The AuSSA is “Australia’s main source of data for the scientific study of the social attitudes, beliefs and opinions of Australians, how they change over time, and how they compare with other societies” [67]. We previously included questions in the 2003 AuSSA [68] regarding Australians’ knowledge of and views about genetics and the use of genetic information in insurance. A module of questions will be included in the 2021 AuSSA to assess participants’ awareness and understanding of the moratorium; views regarding the use of genetic test results by life insurance companies; and the effect of insurance implications and the impact of the moratorium on their desire to undergo genetic testing in future. Questions included in the 2003 survey which remain relevant will be included again to allow for comparison. The demographic data collected by the AuSSA will enable comparisons based on income, education and other pertinent factors.

#### Part 2: health professionals

For the purposes of the A-GLIMMER project, health professionals (HPs) include any qualified health professional who has direct contact with patients who are considering genetic testing. This includes HPs working in genetics services, such as genetic counsellors and clinical geneticists, as well as other non-genetic HPs who discuss genetic testing with patients, such as nurses and oncologists.

Part 2 is designed to address the following objectives:

- **OBJECTIVE 2.1** Assess the level of understanding of the moratorium by health professionals.
- **OBJECTIVE 2.2** Describe experiences of health professionals regarding the impact of the moratorium on patients.
- **OBJECTIVE 2.3** Describe health professionals’ views on regulation and the moratorium’s effectiveness

In Australia, some opportunistic data collection from interviewing health professionals occurred as part of a project which aimed to verify reports of GD by consumers [69], but did not systemically collect views and experiences of health professionals. Prior to the commencement of the moratorium, members of the A-GLIMMER research team conducted the first dedicated survey of Australian health professionals to understand their views and experiences regarding the use of genetic test results in life insurance underwriting [70]. This research focussed on health professionals working in a clinical genetics context (n=87), who observed that many patients needed time to reconsider testing once insurance implications are raised, and some subsequently chose to delay testing or never return. This is consistent with research showing fear of insurance consequences can deter pursuit of genetic testing and participation in genetic research, even where interventions following a positive result can significantly reduce morbidity and mortality [7–9]. In line with the relevant professional guideline [52], genetic professionals in Australia reported almost always discussing life insurance with individuals who are considering genetic testing [70], making an adequate understanding of these issues critical.

During the data collection period for the pre-moratorium survey, some feedback was obtained regarding the questions asked and the process of completing the survey. Prior to finalising this protocol, the proposed follow-up survey questions were piloted on several genetics professionals in different roles, who provided feedback about content, clarity and flow.

##### Health professionals survey—Objectives 2.1 and 2.2

Following commencement of the moratorium, health professionals who discuss genetic testing with patients will be invited to participate in an online survey (see Additional file 1). Because the recruitment criteria has been extended beyond only genetics professionals working in genetics services, we expect that the number of participants will exceed that of the previous survey [70]. Recruitment will be supported by partner organisations including the Human Genetics Society of Australasia, Australian Genomics, and other groups with links to HPs, as well as social media advertisements, direct email to professional contacts of the research team, and snowballing. Questions will be asked regarding HPs’ level of understanding of the moratorium, experiences regarding the impact of the moratorium on patients, and views on regulation of use of genetic test results in underwriting. Results will be compared with the previous research described above [70] to capture changes over time. Participants who complete the online survey will be given the choice to remain anonymous or to provide their details and consent to being contacted for a follow-up interview. Those who provide consent will participate in a semi-structured interview of approximately 20 minutes’ duration, to explore in greater depth their responses to the survey questions. These interviews will be transcribed and analysed qualitatively using thematic analysis.

#### Part 3: genetic researchers

For the purposes of A-GLIMMER, genetic research is research that is done with respect to human genetics and genomics. This refers to research projects in which individuals sign up as research participants, provide samples for DNA analysis and receive a result.

Part 3 is designed to address the following objectives:

- **OBJECTIVE 3.1** Assess the impact of the moratorium on the conduct of genetic research
- **OBJECTIVE 3.2** Assess the impact of the moratorium on genetic research participants

Prior to finalising the protocol, feedback was sought from several prominent genetic researchers regarding their potential willingness to be involved in, and the perceived value of, this research. Genetic researchers indicated through this process that this was an area of concern, that gathering these views would be beneficial, and that there was strong interest in being interviewed for this purpose.

##### Researcher interviews—Objectives 3.1 and 3.2

Previous research has demonstrated the impact of insurance implications on research participants’ willingness to be involved in genomic research, especially where results of clinical significance may be returned to participants [7–9]. In one study, the number of people who declined predictive testing when informed of the insurance implications was more than double the number who declined without knowledge of the insurance implications [8]. Each of these studies collected this data as part of a broader research study, rather than designing the study for the purpose of considering the impact on research of insurance implications and regulatory change. Part 3 of A-GLIMMER’s post-moratorium study will focus on this impact on research studies.

Researchers who conduct research related to human genetics will be interviewed to explore the impact of the moratorium on conducting genetic research and participation in genetic research. Australian researchers who have significant responsibility in leading large genetic research studies will be invited by email to take part in the study. A list of eligible researchers will be identified collaboratively through input from research partners and partner organisations who are aware of research being conducted in this space. We estimate that we will be able to identify at least 10-12 researchers who fit the criteria and expect a response rate of 80%. Data will be captured on the impact of the moratorium on conducting genetic research, including questions about the experience of recruiting; of informing participants about life insurance; the impact that this had on participation rates and individual participants; ethics committee processes; and their views on any changes that they have seen post-moratorium.

#### Part 4: financial industry

Although some individuals apply directly to life insurance companies either by filling out a paper application form or online, many Australians engage a financial adviser/financial broker for advice on and practical assistance with applying for life insurance coverage. It is important to gauge not only the perspectives of the life insurance companies themselves, but also to assess the level of awareness and understanding of the industry professionals who are providing advice to consumers.

Part 4 is designed to address the following objectives:

- **OBJECTIVE 4.1** Assess awareness and levels of understanding of the moratorium by financial industry personnel
- **OBJECTIVE 4.2** Assess the (industry perceived) impact of the moratorium on the Financial Services Industry
- **OBJECTIVE 4.3** Assess the level of adherence to the moratorium by life insurance companies

Before the protocol was finalised, a meeting was held with key underwriting representatives from several of the large Australian life insurance providers to seek feedback regarding the proposed methodology, target groups, and subject matter of interviews.

##### Telephone survey of financial advisors—Objective 4.1 and 4.3

The Australian government publishes a list (n ~ 18,000) of registered Australian financial advisers. Financial advisers will be randomly selected (ensuring a spread across different states of Australia) and invited to complete a short anonymous telephone survey, to assess the understanding of financial industry personnel who are not part of a life insurance company. Participants will be asked questions relating to their knowledge and understanding of the existence and terms of the moratorium.

##### Application form analysis—Objective 4.3

Application forms (pdf or online, depending on availability) will be collected from all underwriters offering risk-rated life insurance in Australia. Content analysis will be conducted to determine whether the forms comply with the terms of the moratorium. Specifically, fields considered will include those seeking information from applicants about past or future genetic testing, and explanation (if any) of the terms of the moratorium. Previous research conducted in 2003 [71] collected and analysed application and personal statement forms from 21 life insurance underwriters. This analysis revealed considerable variation in the genetic information requested by different underwriters in the different forms, and will be compared with the post-moratorium analysis where possible.

##### ***FSC Underwriters survey/interview***—***Objective 4.1, 4.2, and 4.3***

Underwriting representatives from FSC member life insurance companies will be invited to participate in semi-structured interviews or focus groups to explore their views on the moratorium, changes to practice, benefits and limitations, and adherence to terms. Focus groups and interviews will be conducted by videoconference and facilitated by members of the research team. Sixteen life insurance companies are currently members of FSC and it is expected that approximately 10–15 underwriters will attend either a focus group or take part in an interview.

##### ***FSC database analysis***—***Objective 4.3***

The FSC requires its member companies to record in a dedicated database de-identified information regarding all applications for a life insurance product where a genetic test result has been disclosed, either voluntarily or inadvertently [19]. Previous analyses have been conducted on data collected in this database [46, 72]. The FSC, as a study partner, has made changes to the database fields to take into account the different data collection required following the commencement of the moratorium. Data will be extracted annually following the end of financial year, and analysed to assess the volume of applications where genetic test results are disclosed and adherence to the moratorium by insurance companies, and compared with pre-moratorium data where possible.

### Data analysis, regulatory evaluation report and recommendations

Results from each arm of the study will be analysed and published in peer-reviewed journals as they become available.

At the end of the study term (3 years) a Regulatory Evaluation Report will be prepared. The Regulatory Evaluation Report will synthesise the evidence gathered in each arm of the study and use the CMO framework to evaluate the extent to which the moratorium, as implemented by the FSC Standard, has achieved the outcomes intended by the PJC recommendations. The Regulatory Evaluation Report will identify any outcomes that have not been achieved and will draw on the collected data to provide possible reasons why this has occurred. The Report will make recommendations to rectify any failings in relation to the moratorium and to enhance its operation in the future. Consequently, this research project and the Regulatory Evaluation Report will provide valuable evidence toward, although it will not replace, the FSC’s review of the moratorium [14]. The report will also to contribute to fulfilling the PJC’s recommendation that the moratorium be reviewed after five years [13]. The Regulatory Evaluation Report will be provided to the Treasurer and the Minister for Health, the Secretaries of their respective Departments, and the Chair of the PJC. The Report’s recommendations will provide the basis on which future arrangements for the moratorium, or requirements for further regulatory intervention, can be determined and implemented with all relevant decision-makers and stakeholders.

## Discussion

Our project brings together Australia’s leading researchers, clinicians, patient groups, policy experts and industry representatives to answer an over-arching research question*—to what extent does the self-regulated FSC moratorium achieve the aims of addressing concerns with GD as identified by the Parliamentary Joint Committee?*

Strengths of the study include an experienced and diverse investigator group from across Australia that has published extensively together in the area [1, 34, 70, 73–78], and built upon previous research over two decades from some of the group members [8, 42, 46–48, 77, 79–84]. The project was made possible by an Australian government grant which was endorsed by the Victorian Department of Health & Human Services, Human Genetics Society of Australasia and over 20 other project partners, reflecting its widespread support and significance. A key partner is the Financial Services Council (FSC), which represents and facilitates collaboration with members of the Australian life insurance industry. FSC’s willingness to partner with the project and provide collaborative input strengthens the research potential and signifies FSC’s commitment to this important issue. The project is aligned with Australian Genomics, a national collaborative research partnership of more than 80 organizations piloting a whole-of-system approach to integrating genomics into healthcare [85]. The project is also aligned with international efforts, with engagement from several comparable groups in Canada, USA and UK.

The study has limitations and risks which must be acknowledged. The diverse methods of data collection being undertaken across the four stakeholder groups could be challenging to synthesise in a final report. The study may be more likely to collect data from highly motivated or vocal stakeholders, rather than a truly representative cross-section of the community. Further, there is a risk of investigator team bias, given individual views on the issue of GD. We have taken deliberate steps to mitigate against these risks, to ensure rigour and objectivity in our study.

The study’s limited timeline presents another challenge, given the broad and diverse scope of work to be completed. Various challenges or delays could prevent key milestones from being achieved. For example, difficulties in recruiting participants, or obtaining necessary ethics approvals, could influence the planned timeline and milestones. Further disruptions caused by the Covid-19 pandemic may also create challenges for recruitment and data collection. Other risks for the study include the availability of industry-collected data. As study partners, FSC has pledged to provide access to certain industry data, but the research team does not have primary access to this data, and so it is possible that access to this data could be delayed or inconsistent.

In conclusion, the findings of this study will provide valuable evidence to inform the FSC review of the moratorium in 2022, and future policy regarding the use of genetic information in life insurance.

## Supplementary Information

**Additional file 1**. Title of data: Health professional questionnaire. Description of data: questionnaire to be administered to health professionals after the introduction of the moratorium

## Data Availability

No data is included in the manuscript.

## Abbreviations

A-GLIMMER: The Australian Genetics and Life Insurance Moratorium—Monitoring the Effectiveness and Response
CMO: Context-mechanism-outcome
ELSI: Ethical, legal and social issues
FSC: Financial Services Council
GD: Genetic Discrimination
GINA: Genetic Information Nondiscrimination Act (US)
GNA: Genetic Nondiscrimination Act (Canada)
HP: Health professional
PJC: Parliamentary Joint Committee
